# A New Insight on the Radioprotective Potential of Epsilon-Aminocaproic Acid

**DOI:** 10.3390/medicina56120663

**Published:** 2020-11-30

**Authors:** Timur Saliev, Dinara Baiskhanova, Dmitriy Beznosko, Dinara Begimbetova, Bauyrzhan Umbayev, Talgat Nurgozhin, Ildar Fakhradiyev, Baimakhan Tanabayev, Dainius Pavalkis

**Affiliations:** 1 S.D. Asfendiyarov Kazakh National Medical University, Almaty 050000, Kazakhstan; talgat-nur@mail.ru (T.N.); ildariko@mail.ru (I.F.); 2 National Laboratory Astana, Nazarbayev University, Nur-Sultan 010000, Kazakhstan; dinara.baiskhanova@gmail.com (D.B.); dinara.begimbetova@nu.edu.kz (D.B.); bauyrzhan.umbayev@nu.edu.kz (B.U.); 3 Clayton State University, Morrow, GA 30260, USA; dmitriybeznosko@clayton.edu; 4 South-Kazakhstan Medical Academy, Shymkent 160012, Kazakhstan; b.tanabayev@mail.ru; 5 NJSC “Astana Medical University”, Nur-sultan 010000, Kazakhstan; pavalkis.d@amu.kz

**Keywords:** radiation, protection, epsilon-aminocaproic acid, DNA damage, reactive oxygen species, apoptosis

## Abstract

*Background and objectives:* The aim of the study was to scrutinize the ability of epsilon-aminocaproic acid (EACA) to prevent radiation-induced damage to human cells. *Materials and Methods:* Human peripheral blood mononuclear cells (PBMCs) were exposed to ionizing radiation at three low doses (22.62 mGy, 45.27 mGy, and 67.88 mGy) in the presence of EACA at the concentration of 50 ng/mL. *Results:* EACA was able to prevent cell death induced by low-dose X-ray radiation and suppress the formation of reactive oxygen species (ROS). EACA also demonstrated a capacity to protect DNA from radiation-induced damage. The data indicated that EACA is capable of suppression of radiation-induced apoptosis. Comparative tests of antioxidative activity of EACA and a range of free radical scavengers showed an ability of EACA to effectively inhibit the generation of ROS. *Conclusions:* This study showed that the pretreatment of PBMCs with EACA is able to protect the cells from radiation-elicited damage, including free radicals’ formation, DNA damage, and apoptosis.

## 1. Introduction

Despite recent progress in research on radioprotective compounds, no drugs have been officially approved and implemented worldwide as prophylactic agents against ionizing radiation. Therefore, there is a great need for such agents, especially for patients undergoing radiation therapy, and particularly for medical staff involved in the servicing radiation-emitting devices. Moreover, effective radioprophylactic medicines are essential for the protection of military and civil personnel servicing nuclear reactors. In the context of recent catastrophes at nuclear stations such as the Chernobyl and Fukushima incidents, it is highly crucial to find a safe and reliable remedy for the prevention of lethal pathological effects triggered by ionizing radiation, including hematopoietic, gastro-intestinal, vascular reactions, and DNA damage [1,2,3,4].

Radiation-induced DNA damage can be subclassified into two categories: direct and indirect [5,6,7,8,9]. In fact, the DNA structure can be damaged directly by radiation leading to point mutations, DNA strand breaks, and chromosome aberrations. The indirect action of radiation has been linked to ionization that triggers the formation of chemically highly active and unstable substances such as free radicals and reactive oxygen species (ROS). The main damage induced by X-rays and γ-rays are associated with molecules’ interaction with the hydroxyl radical [5]. It induces DNA single and double breaks and lipid peroxidation of the cell membranes [10,11]. The extent of DNA damage and efficacy of its repair depends on many factors, including dose of radiation, time of exposure, the complexity of the DNA site, level of isolation and position of DNA lesions [11]. The problems with DNA repair can potentially lead to mutations and chromosomal aberrations that, in turn, could trigger carcinogenesis [12].

To date, just a few agents have been officially approved as radioprotective compounds such as amifostine and palifermin [5]. The main indication for amifostine is the reduction of xerostomia (mouth dryness) for patients undergoing radiotherapy for cancer located at the head and neck [13]. Palifermin is prescribed for decreasing the risk of the development of oral mucositis for patients who are undergoing radiotherapy [14]. However, both drugs cannot be considered as full protection against lethal bio-effects mediated by radiation. Moreover, amifostine possesses a high potential toxicity, and its administration can lead to some serious adverse effects such as vasodilation, erythema, fever, allergic reaction, and hypotension [15,16]. Another promising prophylactic agent is aminophosphorothioate (WR-3689), that demonstrated therapeutic potential in experiments with rat spinal cord [17]. However, some studies did not confirm the radioprotective effect of WR-3689 [18,19]. Apart from that, there is a range of other compounds with potential radioprotective properties, but most of them are still in the preclinical study stage [20].

As an alternative to chemical agents, some researchers suggested employing vitamins and natural compounds that might potentially serve as a prophylactic remedy against radiation-induced injury [21]. For example, Cinkilic et al. studied the radioprotective effect of cinnamic acid, a natural phenolic phytochemical obtained from cinnamon oil [22], and chlorogenic and quinic acids as well [23]. Some authors stressed that, despite a high potential of current antioxidants, such compounds still cannot provide full radioprotection or be employed as radioprophylactic agents [24,25]. Citrin et al. pointed out that, although hydroxyl radicals can be equally scavenged by both radioprotectors and antioxidants, cellular and in vivo radioprotection was confirmed only with radioprotectors [24]. It indicates that hydroxyl radicals are mainly responsible for radiation-induced DNA damage. In addition, Citrin et al. also pointed out that less-reactive secondary species such as hydroxyl radicals cannot be blocked by standard antioxidants, such as vitamin C and vitamin E, due to their inability to accumulate in the proximity of secondary radical and low kinetic reactivity.

Our study aimed at investigating the ability of epsilon-aminocaproic acid (EACA) to act as a radiopreventive remedy by suppressing the radiation-induced formation of ROS and DNA damage. EACA is an inhibitor of the plasmin–plasminogen system, and its pharmacological properties have been intensively studied and described in the literature since the 1950s [26,27,28,29,30]. This drug has been acknowledged in the literature as an effective agent to control bleeding. In addition to the proven anti-fibrinolytic action, it has been also demonstrated that EACA is capable of inhibiting the activity of some viruses, including adenoviruses [31,32,33].

For experiments, we utilized human peripheral blood mononuclear cells (PBMCs) taken from healthy volunteers. PBMCs are vital component of the immune system for detecting and eliminating abnormal cells, bacteria, and viruses. Importantly, PBMCs have been considered as most radiosensitive cells [34,35,36,37]. Thus, the protection of healthy PBMCs against ionizing radiation-induced damage is highly crucial for persons exposed to ionizing radiation. In our experiments (in vitro), samples of human PBMCs were subjected to ionizing radiation of three low doses of X-ray radiation (22.62 mGy, 45.27 mGy, and 67.88 mGy) in the presence of EACA.

Damaging impact of low-dose ionizing radiation on DNA of human and animal cells has been a topic of many studies [38,39,40,41,42,43,44,45]. It was found out that in animals, chromosomal aberrations were detected between 50 mGy and 0.1 Gy of low linear energy transfer radiation, while in humans, DNA damage was detected in children who underwent CT scans with an estimated radiation dose as low as 0.15 mGy shortly after CT examination [41]. The problem of the DNA damage induced by low-dose radiation must be taken into account due to the number of patients who are undergoing routine radiographic procedures such as CT, breast mammography, or other methods [46].

In our study, after radiation exposure, PBMCs were collected and analyzed to determine cell viability, level of reactive oxygen species (ROS), DNA damage, and apoptosis. In addition, we compared free radical scavenging activity of EACA versus standard scavengers commercially available on the market.

## 2. Materials and Methods

### 2.1. Design of the In Vitro Study

EACA, linear formula H_2_N(CH_2_)_5_CO_2_H and molecular weight 131.17, was purchased from Sigma-Aldrich Merck (St. Louis, MO, USA). First, EACA powder was dissolved in water at 25 mg/mL concentration. Then, EACA from stock solution was dissolved in RPMI 1640 Medium (Thermo Fisher Scientific, Waltham, MA, USA) to achieve the required concentration of 50 ng/mL, which was found non-toxic for PBMCs.

The cell cultures were subdivided into the following groups: (1) control group (no exposure to radiation); (2) group “cells + hydrogen peroxide (H_2_O_2_)” (no exposure to radiation); (3) group “cells + EACA (50 ng/mL) + hydrogen peroxide” (no exposure to radiation); (4) group “cells + X-ray irradiation 22.62 mGy”; (5) group “cells + EACA (50 ng/mL) + X-ray irradiation for 22.62 mGy”; (6) group “cells + X-ray irradiation for 45.27 mGy”; (7) group “cells + EACA (50 ng/mL) + X-ray irradiation 45.27 mGy”; (8) group “cells + X-ray irradiation 67.88 mGy”; (9) group “cells + EACA (50 ng/mL) + X-ray irradiation 67.88 mGy” (Table 1).

Groups #3, 5, 7, 9 were pre-incubated with EACA for 12 h prior to radiation treatment at 37 °C, 5% of CO_2_ and 85% humidity. Afterwards, cells were treated with X-ray irradiation at the doses of 22.62 mGy, 45.27 mGy, and 67.88 mGy. Treatment with hydrogen peroxide (H_2_O_2_) was used as a positive control of DNA damage and apoptosis. The concentration of hydrogen peroxide was of 0.5 mM (final concentration).

### 2.2. Cell Culture

Peripheral blood mononuclear cells (PBMCs) were isolated from fresh whole blood obtained from both male and female volunteers (two males and two female volunteers aged 28–43 years; all nonsmokers), where samples from each gender were equally represented in each experimental group. The samples were obtained at the Republican Research Center for Emergency Care, Astana, Kazakhstan. All protocols pertaining to human subjects were first approved by the Ethics Committee of the S.D. Asfendiyarov Kazakh National Medical University, Kazakhstan (N13/77, dated 26 December 2018). Whole blood was collected into vacutainers containing ethylenediamine tetraacetic acid (EDTA) (Sigma-Aldrich Merck, St. Louis, MO, USA).

PBMCs were isolated from whole blood samples within 3 h after sample collection. All procedures were conducted under sterile conditions in laminar flow hoods and CO_2_-incubators. The protocol of collection and isolation of PBMCs was previously described by Maguire et al. [47]. Briefly, PBMCs were isolated from heparin-stabilized blood using one-step gradient Histopaque (Histopaque, Sigma, density 1077 g/cm^3^). PBMCs were isolated using the method of centrifugation at 400× *g* for 30 min. Interphase rings of PBMCs were collected using sterile pipette and washed threefold with phosphate buffered saline (PBS, Sigma Aldrich). After each collection, cells were centrifuged at 250× *g* for 10 min. Supernatant was discarded after centrifugation. Washed cells were resuspended by adding 5 mL of RPMI medium.

### 2.3. Cytotoxicity Assay

Peripheral blood mononuclear cells (PBMCs) were seeded in 96-multi-well plates (Corning^®^ Costar^®^ 96-Well Cell Culture Plates (Sigma-Aldrich Merck, St. Louis, MO, USA) and incubated for 24 h. After this, PBMCs were exposed to EACA in the following concentrations: 50, 100, 150, 200, and 250 ng/mL. After 12 h, viable cells were counted by using Cell Counting Kit-8 (CCK-8, Sigma-Aldrich, USA) and Synergy H1 Hybrid Multi-Mode Microplate Reader (BioTek, Winooski, VT, USA). The percentage of viable cells was calculated and compared to the sham group. The same method was utilized for viability studies.

### 2.4. Reactive Oxygen Species (ROS) Measurements

CM-H_2_DCFDA (No. C6827, Life Technologies, Thermo Fisher Scientific, Waltham, MA, USA) was used for the determination of ROS level in cell culture exposed to EACA and X-rays [48,49]. H_2_DCFDA was diluted in 34.6 µL of dimethyl sulfoxide (DMSO). Immediately after exposure, the cells with medium were placed into 96-well plates followed by addition of H_2_DCFDA (10 µL of the dye to 90 µL of cells). The resulted solution was carefully resuspended and incubated at 37 °C for 30 min. After incubation, the level of ROS was measured using Synergy H1 Hybrid Multi-Mode Microplate Reader (BioTek, USA). Hydrogen peroxide (H_2_O_2_), a standard inducer of apoptosis and ROS, was used as a positive control (in final concentration of 0.5 mM). PBMCs were pre-incubated with ethanol (10 mmol/L) for 30 min prior to ROS measurements.

### 2.5. DNA-Comet Assay

The DNA-comet assay was performed immediately after exposure to radiation. The assay was conducted according to Amsbio protocol (Amsbio LLC, Cambridge, MA, USA) with some modifications. Briefly, cells were washed twice in PBS, centrifuged at 700× g for 15 min, and resuspended at 1 × 104 in 30 µL of 1% (w/v) low melting point (LMP) agarose (Sigma-Aldrich). The cell suspension was put over Comet slides with specially treated glass surface (Amsbio), and it was allowed to incubate at 4 °C for 10 min. After this, coverslips were removed and slides were placed into pre-chilled lysis solution (Amsbio) for 60 min at 4 °C or overnight to remove proteins.

Following lysis, slides were placed into a gel electrophoresis camera, and then incubated in fresh alkaline electrophoresis buffer for DNA denaturation (per 1 L of the buffer 30 mL of NaOH and 5 mL of EDTA, taken from stock solutions 10 N NaOH and 200 mM Na2 EDTA, pH = 13) for 20 min at room temperature. After incubation, electrophoresis was performed at 24 V and 300 mA (1 V per 1 cm platform length for slides in electrophoresis camera) for 20 min at room temperature. All procedures were conducted in the dark to minimize DNA damage. Following electrophoresis, the slides were fixed and washed for 10 min to remove alkalis and detergents, and then they were dried in the dark. Slides were stained by SYBR Green dye (Amsbio) for 2 min, covered with cover glass, and dried at room temperature in the dark.

Scoring the DNA damage: Slides were examined at 200× magnification under an Olympus trinocular fluorescence microscope (Olympus BX53, Shinjuku-ku, Tokyo, Japan). Visual and computerized image analyses of DNA damage were carried out by using the TriTek CometScore ™ program (TriTek Corp., Sumerduck, VA, USA). The visual analysis of one hundred randomly selected non-overlapping cells was based on perceived comet tail length migration and relative proportion of DNA in the comet tail. The index “% DNA in the comet tail” was determined by the number of low molecular DNA fragments formed from discontinuities alkali labile DNA segments, and migrated towards the anode by electrophoresis.

### 2.6. Apoptosis Measurement

Annexin V-FITC/PI Apoptosis Detection Kit (Cell Signaling Technology, Danvers, MA, USA) was used for apoptosis detection [50]. After exposure to X-ray radiation, PBMCs were collected and kept for 6 h in the incubator (37 °C). Then, PBMCs were collected and washed with PBS at 4 °C. Then, 1X Annexin V Binding Buffer and Annexin V-FITC conjugate were added to the cell suspension. The early apoptotic PBMCs were identified by direct visualization of Annexin V green-colored membrane staining under an inverted motorized fluorescence microscope Olympus IX83 (Shinjuku-ku, Tokyo, Japan) and an Olympus XM-10 camera (Shinjuku-ku, Tokyo, Japan). DAPI dye (4′,6-diamidino-2-phenylindole, Thermo Fisher Scientific, Waltham, MA, USA) was utilized for cell staining. Cells were analyzed and counted using MetaMorph 7.8 software (Molecular Devices, LLC., San Jose, CA, USA). To distinguish PBMCs that lost their membrane integrity, Propidium Iodid dye (Thermo Fisher Scientific, Waltham, MA, USA) was added in a final concentration of 500 nM before cell counting. DAPI’s excitation spectrum is at 360 nm and emission one is at 460 nm, while Annexin V-FITC’s excitation and emission are 494 nm and 518 nm, respectively. PI has excitation maximum at 535 nm and fluorescence emission maximum at 617 nm.

### 2.7. Tests for Free Radical Scavenging Activity

The comparative tests of anti-ROS activity of EACA and a range of free radical scavengers (antioxidants) was conducted. As an inducer of ROS generation in PBMCs culture, hydrogen peroxide was used in the concentration of 1.0 mM. For the experiments, in vitro effective doses of free radical scavengers according to data from published reports were utilized: *N*-acetyl-cysteine (NAC) 30 nM [51], caffeic acid (30 μg/mL) [52,53], p-coumaric acid (160 μg/mL) [54], gallic acid (70 μg/mL) [55], quercetin (33.8 μg/mL) [56,57], ascorbic acid (80 µg/mL) [58,59], lipoic acid (10.3 µg/mL) [60], polydatin (10 µg/mL) [61], and tocopherol (Vitamin E) (115 µg/mL) [62,63]. The EACA was used in the same concentration as for the main experiments (50 ng/mL) with radiation.

### 2.8. Exposure Set-Up

One day prior to radiation exposure, PBMCs were cultured in a 6-well plate (Corning^®^ Costar^®^ cell culture plates, 6 well, Sigma-Aldrich Merck, St. Louis, MO, USA). Cells (at density 5 × 10^5^ mL) were irradiated with 100 kVp X-rays (Philips, Eindhoven, The Netherlands) at doses of 22.62 mGy, 45.27 mGy and 67.88 mGy in a radiation field of 12 × 12 × 13 cm. The dose rate of X-rays was 0.0226 Gy/min. The distance between the source and surface of the plate was 30 cm. After irradiation, PBMCs were analyzed for cell viability, ROS production, apoptosis, and DNA damage according to the abovedescribed protocols. The unexposed group of PBMCs was used as a control.

### 2.9. Radiation Dosimetry

Calibrated radiochromic EBT3 films (Gafchromic EBT, Ashland Advanced Materials, Bridgewater, MA, USA) were employed for radiation dosimetry. The films were cut and placed below the plate in order to detect the planar dose deposition. The detected doses were calculated and compared to the doses predicted by calculation from treatment planning data for the kVp beams. Such a method of radiation dosimetry was chosen due to its optimized planar geometry and good spatial resolution that facilitates the calculation of the dose expected at the cell layer for each well. The method was previously described by Mackonis et al. [64].

### 2.10. Statistical Analysis

For the analysis of the impact of ionizing radiation on cell viability, ROS production, DNA damage, and apoptosis induction in human PBMCs, one-way factorial analysis of variance (one-way ANOVA with replication) was utilized. The analysis was used for each of the tested variables. The Tukey test (‘honestly significant difference’ (HSD) for unequal) was used to test paired differences (for individual groups). Experimental data are represented as means from at least 3 independent samples and the respective standard error of the mean.

## 3. Results

### 3.1. Evaluation of Cytotoxicity of EACA

**Figure 1:**
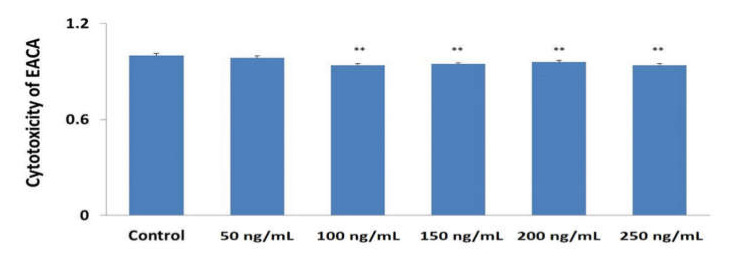
Cytotoxic effect of various doses of epsilon-aminocaproic acid (EACA) on cells viability presented as a normalized ratio to the control. Error bars in the graphs indicate the standard error of the mean (SEM) for *n* = 3 independent experiments. **—*p* ≤ 0.01 compared to the control.

We did not observe a significant decrease in cell viability as a result of an increase in EACA concentration. Nevertheless, the minimal cell cytotoxicity was observed for the concentration of 50 ng/mL (*p* < 0.115 vs. control) (Figure 1). This dosage was chosen for in vitro experiments.

### 3.2. Effect of EACA on Cell Recovery after X-ray Exposure and Treatment with Hydrogen Peroxide

**Figure 2:**
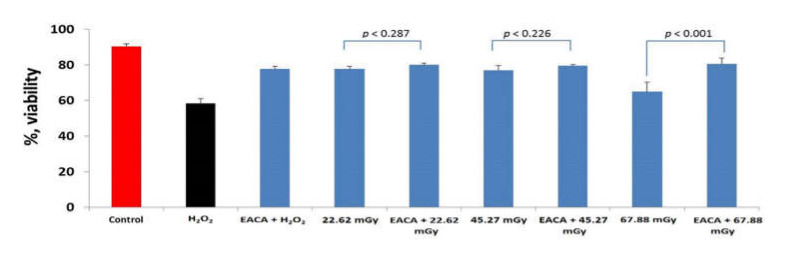
Effect of epsilon-aminocaproic acid (EACA) on cell viability after X-ray exposure (presented as %). The measurements have been done 12 h after exposure to radiation. Normal peripheral blood mononuclear cells (PBMCs) were subjected to three radiation doses: 22.62 mGy, 45.27 mGy, and 67.88 mGy in the presence of EACA (50 ng/mL). Error bars in the graphs indicate the standard error of the mean (SEM) for *n* = 3 independent experiments.

PBMCs were treated with X-ray radiation with and without the presence of EACA (50 ng/mL). In the group of the positive control (hydrogen peroxide), we observed a significant reduction of cell viability (*p* < 0.001 vs. control) (Figure 2). At the same time, we observed a decrease in the number of viable cells in the groups subjected to low-dose X-ray radiation. However, the maximum cell death was detected in the group irradiated by the highest radiation dose 67.88 mGy (*p* < 0.001). The data showed that the pretreatment of PBMCs with EACA has prevented cell loss in the groups subjected to radiation. However, ANOVA analysis demonstrated that there is no statistical difference between groups exposed to the lowest radiation level (22.62 mGy), and the group treated with the same dose, but with added EACA (*p* = 0.287). Secondly, we did not observe the significant differences in cell loss in the groups subjected to 22.62 and 45.27 mGy. It may indicate that the chosen radiation doses were too low, and they did not noticeably affect cell viability in both groups.

We found a similar statistical difference in the groups ‘X-ray 45.27 mGy’ and ‘X-ray 45.27 mGy + EACA’ (*p* = 0.226). At the same time, EACA was able to prevent cell loss in the group exposed to the highest radiation dose (67.88 mGy) (*p* < 0.001). It demonstrated the capability of EACA to protect pretreated cells from ionizing radiation.

### 3.3. Impact of EACA on Production of Reactive Oxygen Species (ROS)

**Figure 3:**
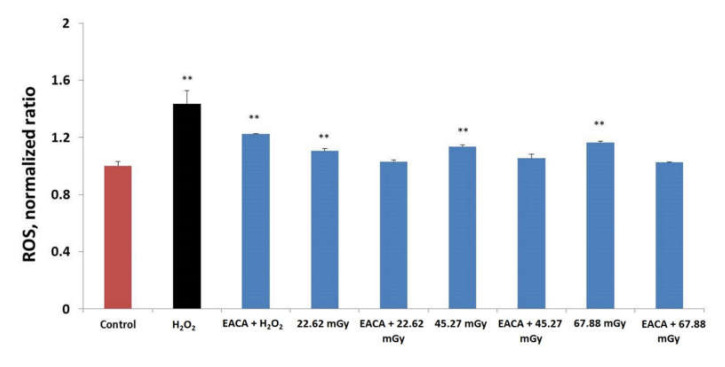
Impact of epsilon-aminocaproic acid (EACA) on production of reactive oxygen species (ROS) presented as a normalized ratio to the control. The measurements were done immediately after exposure to X-ray radiation. Normal peripheral blood mononuclear cells (PBMCs) were subjected to three radiation doses: 22.62 mGy, 45.27 mGy, and 67.88 mGy in the presence or absence of EACA (50 ng/mL). Error bars in the graphs indicate the standard error of the mean (SEM) for *n* = 3 independent experiments. **—*p* ≤ 0.01 compared to the control.

Our data showed that the exposure of PBMCs culture to low-dose X-ray radiation led to the immediate production of intracellular ROS (Figure 3). The maximum of ROS production was detected for 67.88 mGy (*p* < 0.001 vs. control). A comparison of the control group and groups exposed to radiation demonstrated that the pretreatment with EACA resulted in the inhibition of ROS production. EACA slightly lowered ROS levels in all three groups. However, no significant statistical difference was observed. 

### 3.4. EACA and Radiation-Induced DNA Damage

**Figure 4:**
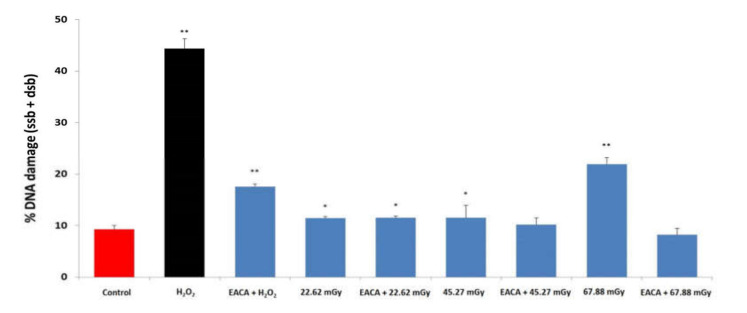
Effect of epsilon-aminocaproic acid (EACA) on DNA damage induced by radiation (%). The measurements were done immediately after exposure to X-ray radiation. Normal peripheral blood mononuclear cells (PBMCs) were subjected to three radiation doses: 22.62 mGy, 45.27 mGy, and 67.88 mGy in the presence or absence of EACA (50 ng/mL). DNA damage is presented as a percentage (%). Error bars in the graphs indicate the standard error of the mean (SEM) for *n* = 3 independent experiments. **—*p* ≤ 0.01 compared to the control; *—*p* ≤ 0.05 compared to the control.

**Figure 5:**
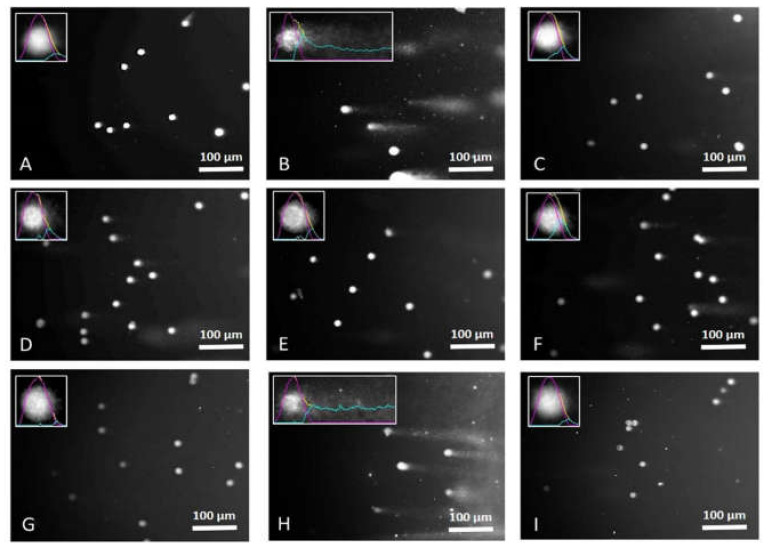
Images of DNA comets of PBMCs subjected to X-ray radiation in the presence or absence of EACA (50 ng/mL). The DNA-comet assay was performed immediately after exposure to radiation. (**A**) DNA comets of control group; (**B**) DNA comets of cells subjected to hydrogen peroxide (positive control); (**C**) DNA comets of cells subjected to hydrogen peroxide and EACA; (**D**) DNA comets of cells exposed to 22.62 mGy X-ray; (**E**) DNA comets of cells exposed to 22.62 mGy X-ray and EACA; (**F**) DNA comets of cells exposed to 45.27 mGy X-ray; (**G**) DNA comets of cells exposed to 45.27 mGy X-ray and EACA; (**H**) DNA comets of cells exposed to 67.88 mGy X-ray; (**I**) DNA comets of cells exposed to 67.88 mGy X-ray and EACA.

The results of alkaline comet assay demonstrated an increase of the percentage of DNA damage (single- and double-stranded DNA breaks) correlating with the elevation of radiation dose (Figure 4 and Figure 5). The highest extent of DNA damage was detected in the group exposed to radiation dose 67.88 mGy (21.93 ± 1.14%; *p* < 0.001 vs. control). EACA showed an ability to prevent radiation-induced DNA single and double-stranded breaks in PBMCs. The introduction of EACA was able to completely diminish DNA damage in the group ‘67.88 mGy + EACA’ to the level of the control group (8.20 ± 1.11%; *p* = 0.316 vs. control, and *p* ≤ 0.001 vs. ‘67.88 mGy’ group) (Figure 4).

These data are in the line with abovementioned studies of cell viability, where cell loss was approximately similar for the groups subjected to 22.62 and 45.27 mGy radiation (but it was significant in the group exposed to 67.88 mGy). It might indicate that doses 22.62 and 45.27 mGy are not strong enough for critical DNA damage.

### 3.5. EACA and Radiation-Induced Apoptosis

**Figure 6:**
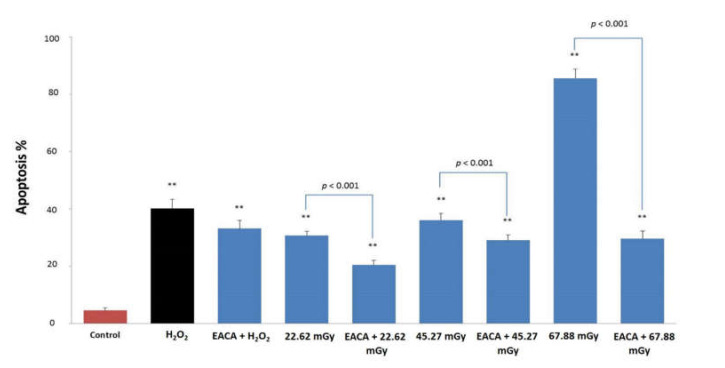
Effect of epsilon-aminocaproic acid (EACA) on the prevention of radiation-induced apoptosis (%). The measurements were done 6 h after exposure to X-ray radiation. Normal peripheral blood mononuclear cells (PBMCs) were subjected to three radiation doses: 22.62 mGy, 45.27 mGy, and 67.88 mGy in the presence or absence of EACA (50 ng/mL). The number of apoptotic cells (Annexin V labeling) is presented as a percentage (%). Error bars in the graphs indicate the standard error of the mean (SEM) for *n* = 3 independent experiments. **—*p* ≤ 0.01 compared to the control.

Pretreatment of PBMCs with EACA resulted in significant suppression of radiation-induced apoptosis (Figure 6). Similar results were detected in the group of the positive control (hydrogen peroxide). The increase in the number of cells in the early apoptotic phase is correlated with a rise of radiation dose, where the maximum was found in the group irradiated with 67.88 mGy (85.60 ± 0.032%; *p* < 0.001 vs. control). The counting apoptotic cells data indicated that EACA is capable of apoptosis inhibition even in the group irradiated with the 67.88 mGy dose, the highest in our experiments (level of apoptosis 29.67 ± 0.025%; *p* < 0.001 vs. control).

**Figure 7:**
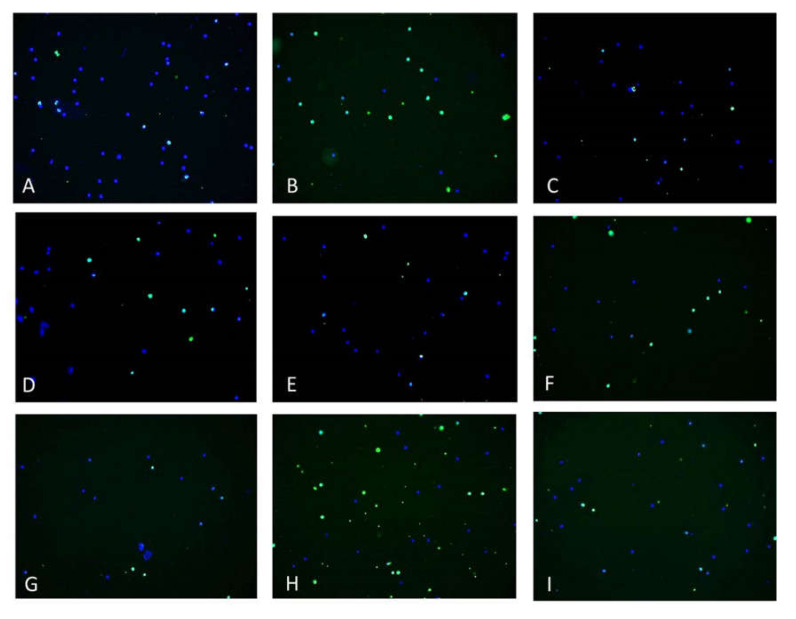
Microscopic images of apoptotic PBMCs (600×). The measurements were done 6 h after exposure to radiation. (**A**) control group; (**B**) cells subjected to hydrogen peroxide (positive control); (**C**) cells subjected to hydrogen peroxide and EACA; (**D**) cells exposed to 22.62 mGy X-ray; (**E**) cells exposed to 22.62 mGy X-ray and EACA; (**F**) cells exposed to 45.27 mGy X-ray; (**G**) cells exposed to 45.27 mGy X-ray and EACA; (**H**) cells exposed to 67.88 mGy X-ray; (**I**) cells exposed to 67.88 mGy X-ray and EACA. Staining by Annexin V (green color) indicates early apoptosis stage; double staining by Propidium Iodide (red) and Annexin V (green) reflects the late apoptosis phase.

Microscopic analysis showed a prevalence of cells that underwent early apoptosis stage (stained with Annexin V dye) in the groups exposed to low-dose X-ray radiation (Figure 7). In addition to predominant green staining, we also observed main morphological signatures of apoptosis such as cell budding, swelling, the formation of “apoptotic body,” and rupture of cells. The maximal number of cells stained by Annexin V was observed in the group exposed to the highest radiation dose of 67.88 mGy (Figure 7H).

### 3.6. Comparison of Anti-ROS Activity of EACA and Free Radical Scavengers

**Figure 8:**
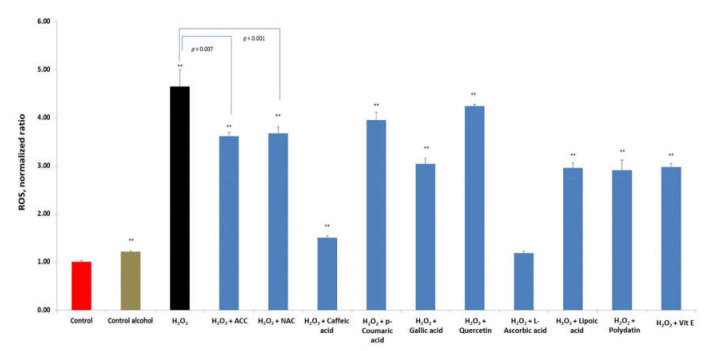
Comparison of anti-ROS activity of EACA (50 ng/mL) and free radical scavengers in the presence of hydrogen peroxide (concentration of 1.0 mM). ROS production is presented as a ratio normalized to the control group. Abbreviations: epsilon-aminocaproic acid (EACA), *N*-acetyl-cysteine (NAC), caffeic acid, p-Coumaric acid, gallic acid, quercetin, ascorbic acid, lipoic acid, polydatin, and Vit E (tocopherol). Error bars in the graphs indicate the standard error of the mean (SEM) for *n* = 3 independent experiments. **—*p* ≤ 0.01 compared to the control. In addition, statistical comparison between the hydrogen peroxide group and free radical scavengers’ groups have been done.

In addition to the main experiments, we carried out comparative tests of anti-ROS activity of EACA and a range of well-known free radical scavengers (antioxidants) (Figure 8). Hydrogen peroxide was employed as an inducer of ROS generation in PBMCs culture. For the experiments, we used in vitro effective doses of free radical scavengers described in the literature: *N*-acetyl-cysteine (NAC) 30 nM [51], caffeic acid (30 μg/mL) [52,53], p-coumaric acid (160 μg/mL) [54], gallic acid (70 μg/mL) [55], quercetin (33.8 μg/mL) [56,57], ascorbic acid (80 µg/mL) [58,59], lipoic acid (10.3 µg/mL) [60], polydatin (10 µg/mL) [61], and tocopherol (115 µg/mL) [62,63]. The EACA was used in the same concentration as for the main experiments (50 ng/mL). Hydrogen peroxide was utilized to trigger ROS formation in PBMCs culture in the concentration of 1.0 mM that was higher than in the main radiation experiments.

The strongest antioxidant activity was demonstrated by caffeic (normalized ratio to control: 1.50 ± 0.04; *p* < 0.001 vs. control) and ascorbic (1.19 ± 0.04; *p* < 0.001 vs. control) acids. EACA showed a high antioxidant activity as well (3.61 ± 0.08; *p* < 0.001 vs. control and *p* = 0.007 vs. the “hydrogen peroxide” group) (Figure 8). These results were similar to the group pretreated with *N*-acetyl-cysteine (3.67 ± 0.14; *p* < 0.001 vs. control; *p* < 0.001 vs. the “hydrogen peroxide” group).

It must be noted that, for comparative tests, we used EACA at an extremely low dose of 50 ng/mL. This dosage is significantly lower than the reported minimal “in vitro effective” plasma concentrations of EACA (44 mcg/mL for infants; and 94 mcg/mL for adults) [65]. These findings indicate that the antioxidant dosage and efficacy of EACA could be increased without possible adverse effects.

## 4. Discussion

Despite an extensive search for safe and effective radioprotectors over the last decade, there is still no ideal drug candidate which can be used for the prevention of radiation-induced lethal effects as a prophylactic agent (i.e., for administration prior to radiation exposure). So far, only two drugs, amifostine and palifermin, have been approved by the United States Food and Drug Administration (FDA). However, their applications have been narrowed to decreasing the radiation-associated symptoms such as “syndrome of dry mouth,” but not for actual radioprotection.

Recent progress in the understanding of mechanisms of cell reaction upon exposure to radiation has led to the exploration and development of different compounds that could serve as radioprotectors. It includes apoptosis modulator Entolimod [66], nitroxide Tempol-H [67], inhibitors of p53 upregulated modulator of apoptosis (PUMA), angiotensin-converting enzyme (ACE) inhibitors, and inhibitors of growth factors and cytokines [5].

Our study aimed at examination of the ability of low doses of epsilon-aminocaproic acid (EACA) to prevent radiation-induced DNA damage on healthy human peripheral blood mononuclear cells (PBMCs). This type of blood cell is a vital part of the immune system to detect and eliminate abnormal cells, bacteria, and viruses. Amongst all types of human cells, PBMCs have been considered as the most radiosensitive ones [34,35,36,37]. So, the protection of healthy PBMCs against ionizing radiation-induced damage is critical for patients undergoing radiotherapy, and for personnel servicing nuclear reactors as well.

In our experiments, we used low doses of radiation (22.62 mGy, 45.27 mGy, and 67.88 mGy) to expose the cells cultured in 6-well plates (Corning Costar^®^ cell culture plate), i.e., at an extremely small effective area (growth area of a 6-well plate is 9.5 cm^2^) compared to whole-body radiation. These doses were applied just for very short periods of time (1, 2, and 3 min, correspondingly) compared to one year of exposure required for the maximum permissible dose (MPD). The average dose we applied was 45.25 mGy. In a range of studies that involved human isolated PBMCs for the investigation effect of low-dose radiation, the average dose was about 50 mGy [47,68,69,70]. Moreover, it was noted that analysis of radiation-induced genetic damage using white blood cells can be useful to estimate doses received by people in whole-body irradiation (“accidental”), where the detection limits correspond to 100 mGy X-ray (Book “The dosimetry of ionizing radiation,” page 270, by Johan J. Broerse et al., Editor Kenneth Kase). From a statistical point of view, the detection threshold for manual scoring of dicentric cells is 0.5 Gy for the analyzed 50 cells, while it is 0.2 Gy for 500 cells. The detection limit for automatic scoring is 0.3 Gy for 1000 cells and 0.2 Gy for 3000 cells [71].

Birnboim and Jevcak have already demonstrated that even low-dose radiation (50 mGy) is capable of inducing DNA strand breaks in human white blood cells [70]. Besides that, there is a significant body of evidence of the cell-damaging effect of low-dose radiation that was related and nonrelated to DNA damage [72,73,74,75,76,77,78,79,80,81]. The topic on the relevance for radiation protection of recent findings in low-dose radiobiology has been intensively discussed in the literature [82,83].

Our study demonstrated that pretreatment of PBMCs with the low dose of EACA (50 ng/mL) led to the prevention of bio-effects induced by low-dose ionizing radiation, including cell loss, free radical formation, DNA damage, and apoptosis (“programmed cell death”). EACA is a well-known inhibitor of the plasmin–plasminogen system intensively used as an effective drug for bleeding control. In addition to its anti-fibrinolytic properties, EACA is capable of suppressing activity of some viruses [31,32,33], indicating that this compound can act as a protease inhibitor. It has been already shown that some protease inhibitors can be effectively employed for radioprotection. For example, Dittmann et al. demonstrated the radioprotective potential of the Bowman–Birk protease inhibitor [84,85].

The capacity of protease inhibitors to decrease apoptosis in white blood cells has been already reported previously in the literature [86,87,88]. The underlying mechanism is mainly associated with the suppression of caspases activity, which is responsible for the apoptotic cascade reactions. However, some authors emphasized the role of inhibition of mitochondrial transmembrane potential and the release of apoptotic mediators from mitochondria, such as cytochrome *c* [88,89]. It must be also noted that the proteases have been considered as a key component of apoptosis initiation [90,91,92,93,94,95]. Therefore, we hypothesize that the protease suppression by the means of EACA can be involved in cell protection from radiation-induced apoptosis.

Apart from the protease activation, the initiation of apoptosis has been also linked to the generation of ROS as a response to external stimuli such as ionizing radiation [96,97,98]. It was shown that the radiation-induced ROS formation leads to the elevation of glutathione level, caspase activation, the release of mitochondrial cytochrome *c*, imbalance of level of mitochondrial NADP^+^-dependent isocitrate dehydrogenase, an increase of concentration of cytosolic Ca^2+^, and, consequently, to the triggering of apoptosis [99,100,101]. Moreover, ROS are involved in the formation of closely clustered DNA lesions and induction of DNA double-stranded breaks [1,102,103]. In our experiments, a low dose of EACA was able to inhibit the generation of ROS and minimize DNA damage. The observed effect might be associated with activating cellular antioxidant systems such as glutathione (GSH) [104]. GSH consists of three residues (γ-l-glutamyl-l-cysteinyl glycine) which can effectively scavenge intracellular ROS either directly or indirectly [105]. It was shown that GSH can directly neutralize O−∙2 and other types of ROS. In addition, GSH is able of revitalizing other cellular antioxidant systems and stabilizing cellular membrane. Apart from GSH, EACA could be implicated in the activation of the sulfaredoxin and thioredoxin antioxidant systems, which are responsible for maintaining peroxiredoxins, enzymes with high catalytic activity against intracellular ROS [104,106,107]. Previously, Mongia et al. demonstrated that protease inhibitors inhibit the apoptosis and oxidative stress [108]. The authors hypothesized that protease inhibitors may either be neutralizing the ROS-induced oxidative stress or decreasing the generation of ROS.

Our results indicated that pre-incubation PBMCs with EACA did not affect apoptosis from exposure to H_2_O_2_; however, it does suppress the development of apoptosis induced by ionizing radiation. The data of some studies showed that hydrogen peroxide mainly induces apoptosis via mitochondrial pathway [109], mitochondrial permeabilization, and regulating mitogen-activated protein kinase (MAPK) signaling pathways [110]. Cao et al. demonstrated that ionizing radiation selectively induces the activation of caspase-9 and caspase-3/7, but not caspase-8, by triggering mitochondrial outer membrane permeabilization (MOMP) [111]. Moreover, it was found that both pro-apoptotic and anti-apoptotic Bcl-2 family proteins were involved in radiation-induced apoptotic signaling pathways. Cao et al. also hypothesized that ionizing radiation specifically triggers the intrinsic apoptotic signaling pathway through Bcl-2 family protein-dependent mitochondrial permeabilization. Taking into account the abovementioned, the capacity of EACA to block radiation-induced apoptosis can be linked to the suppression of specific caspases. However, this topic requires further extensive studies and validation.

In addition to the main studies on radioprotective efficacy of EACA, we have also compared the antioxidant activity of EACA and commercially available free radical scavengers. Most of them have been routinely used for bio-chemical experiments for many years worldwide. Moreover, natural antioxidants have become a vital part of the anti-aging diet and therapy. We employed doses that have been reported in the literature as effective in vitro. Our findings indicate that the EACA in low concentration is capable of suppressing the formation of ROS (induced by hydrogen peroxide). The results obtained were similar to the group pretreated with *N*-acetyl-cysteine (NAC). Despite these encouraging results, we believe that more extensive studies towards comparing various doses of EACA and other antioxidants would shed light on the real potential of EACA as a novel free radical scavenger.

In fact, the ionization of molecules caused by radiation induces the generation of free radicals responsible for DNA damage and oxidation of vital cellular structures. However, human cells demonstrate different radiosensitivity [112,113]. It has been thought that fast-dividing cells are more susceptible to ionizing radiation. In this regard, the most sensitive to radiation-induced DNA damage are PBMCs presented mainly by lymphocytes and monocytes. The radioprotection of this type of blood cells is a highly important task for national health services, particularly for the protection of cancer patients routinely undergoing radiotherapy as a vital component of anticancer treatment. Moreover, the antiviral potential of EACA would be also beneficial for application in oncological clinics [32,114]. It is well known that chemotherapy causes the suppression of the immune system, thus increasing the risk of viral infections that predominantly attack the respiratory tract [115,116]. In this regard, the synergetic activity of EACA against adenoviruses (protease-containing viruses) and radiation-induced effects could be harnessed for cancer management.

Aside from cancer treatment, there is also a high demand for the effective pharmaceutical agent for the prevention of radiation-mediated tissue toxicity amongst personnel servicing nuclear reactors/stations. Moreover, such a drug would be beneficial for astronauts, especially for those who participate in long spaceflights, including International Space Station (ISS) missions [117,118]. A sensitivity of the PBMCs of astronauts to double-stranded DNA breaks induced by gamma-radiation has been already reported in the literature [119]. In a broader sense, safe and effective radioprotector could be potentially utilized for the protection of the public in regions exposed to nuclear accidents.

## 5. Conclusions

To summarize, the data of our study indicated the potential of EACA for the prevention of DNA damage in human white blood cells induced by low-dose radiation. We demonstrated that EACA was capable of preventing cell death induced by low-dose X-ray radiation and suppressing the formation of ROS. In addition, EACA showed a capacity to protect DNA from the damage and apoptosis induced by the X-ray radiation. Moreover, the comparative tests of antioxidative activity of EACA and a range of free radical scavengers demonstrated an ability of EACA to inhibit the generation of ROS. However, it must be noted that the presented results are relevant for low-dose radiation only. Thus, the potential of EACA to protect cells and organisms from high-dose radiation needs to be determined in future research.

## Figures and Tables

**Table 1:** Experimental groups.

1	control group (no exposure to radiation)
2	group “cells + hydrogen peroxide (H_2_O_2_)” (no exposure to radiation)
3	group “cells + EACA (50 ng/mL) + H_2_O_2_” (no exposure to radiation)
4	group “cells + X-ray irradiation 22.62 mGy”
5	group “cells + EACA (50 ng/mL) + X-ray irradiation for 22.62 mGy”
6	group “cells + X-ray irradiation for 45.27 mGy”
7	group “cells + EACA (50 ng/mL) + X-ray irradiation 45.27 mGy”
8	group “cells + X-ray irradiation 67.88 mGy”
9	group “cells + EACA (50 ng/mL) + X-ray irradiation 67.88 mGy”

Abbreviations: EACA: epsilon-aminocaproic acid; H_2_O_2_: hydrogen peroxide.
